# Properties of the Portuguese version of the empowerment scale with mental health organization users

**DOI:** 10.1186/1752-4458-8-48

**Published:** 2014-11-25

**Authors:** Maria Fátima Jorge-Monteiro, José Henrique Ornelas

**Affiliations:** ISPA - Instituto Universitário, Rua Jardim do Tabaco, 34, 1149-041 Lisboa, Portugal

**Keywords:** Empowerment scale, Reliability, Validity, Mental health

## Abstract

**Background:**

This study examines the reliability and validity of the Portuguese version of the Empowerment Scale (ES) to be used in the community/psychosocial mental health field. Authors also reviewed the properties of the development and cross-cultural adaptation of the ES. Because mental health services are required to encourage empowerment and recovery-oriented interventions, adequate empowerment-oriented outcome measures are needed to evaluate services and study interventions across countries.

**Methods:**

The current research was part of a larger research project with 213 participants. A confirmatory factor analysis (*CFA*) was conducted to observe the ES’s construct-related validity, and a reliability analysis for internal consistency. The ES concurrent validity with the recovery and psychiatric symptoms measures was also assessed using the Pearson’s correlation coefficient.

**Results:**

The CFA supported the five-factor configuration for the refined model of measure as satisfactory. The Portuguese version of the ES presented an overall satisfactory reliability (α = .79) and was positively correlated with personal recovery (*r* = .71) and inversely with psychiatric symptoms (*r* = -.22).

**Conclusions:**

The overall scale was considered reliable and valid to be used by Portuguese researchers and practitioners to evaluate empowering interventions in mental health services. Furthermore, in the effort to increase ES construct-related validity, this article suggests further improvements to enhance the empowerment measure.

## Background

Nowadays, mental health services in the community are required to develop empowerment and recovery-oriented approaches that challenge traditional structures and processes in mental health practice [1, 2]. This transformation is intended to facilitate community integration, recovery, and to strengthen the participation, as well as the social and political power of people with mental health issues that use those services [3–6].

Community-based research suggested that community and organizational participation fosters the developmental process of personal, social and civic empowerment [7]. Research conducted by consumer-run organizations also revealed increased personal empowerment from participating in strength-based challenging initiatives [8–10].

In the mental health field the concept of empowerment was introduced by the mutual help and advocacy movement [4] and also studied in community psychology [11]. Definitions of empowerment address the multidimensionality and the multi-level aspects of concept as it comprises individual, social and political components [12, 13], whether referring to individuals, groups or communities [14].

Previously published accounts [15], considered it a continuous process of individual development of personal capacity and of community participation: individuals have awareness, optimism for the future, and confidence about decisions, thus revealing agency, and the ability to contribute to collective goals. Authors such as MA Zimmerman and J Rappaport [16] or KI Maton and AE Brodsky [7] viewed empowerment as a principle for action involving participation in groups, increased individual’s control over their life-course and the potential to access and to change community resources.

MA Zimmerman and S Warschausky [17] reviewed a number of studies in rehabilitation literature revealing increased skills and awareness which improve individuals’ sense of control and participation in other community activities. Those studies supported the idea that the core component of empowerment of exerting control over one’s life is a vital step towards improving individual-level outcomes in rehabilitation. Mental health systems are required to develop opportunities for people’s participation in decision-making and in service policy and evaluation.

Adopting an empowerment approach also implies the development of appropriate and valid outcome measures to evaluate practice in similar mental health settings, accordingly. The Empowerment Scale (*ES*) [18, 19], also known as “Making Decisions Scale”, is one of the few existing empowerment outcome measures in the mental health field and therefore widely used and validated across countries and contexts [20–25] to evaluate the efficacy of interventions.

In Portugal, parallel to being the de-institutionalization objective, the reformed mental health policy (Plano Nacional para a Saúde Mental (PNSM) - 2007–2016) created a new law (DL 8/2010) for the implementation of integrated care in the community context, which reviewed a previous framework (DC 407/98) for psychosocial rehabilitation and community support services in the mental health field. The PNSM states the mental health system must address the need for the development of mental health care in the community, the users’ participation and their involvement in the recovery process and personal achievements (the Portuguese plan can be retrieved from one Health Ministry website at http://saudemental.pt/wp-content/uploads/2011/02/relatorioplanoaccaoservicossaudemental.pdf).

The empowerment concept is a relevant principle to respond to transformative changes in the mental health system [26, 27] with implications for both individual and community quality of life [17]. Based on empirical evidence, community mental health organizations (CMHO) may operate as mediating resources to foster individuals’ empowerment [28–30]. Therefore, the adaptation of the ES to the Portuguese context and language is of relevance in the context of current reform and policy change towards an empowerment and recovery-oriented mental health system.

### Development and adaptation of the ES cross-culturally

ES Rogers, J Chamberlin, ML Ellison and T Crean [18], developed the ES in a participatory study with 261 participants from self-help groups, and established the five-factor structure for the ES: esteem and efficacy, power and powerlessness, optimism and control over the future, community activism and autonomy, and righteous anger. The authors also reported a satisfactory degree of internal consistency (α = .86) for the scale. The ES five-factor solution was further validated [19] in a study with a large number of participants (*N* =1827) from a multi-site consumer-operated services research project. The confirmatory factor analysis identified 3 items to be removed from the ES, in which the model with 28items showed a fair fit, and thus provided a revised 25-item version for the empowerment measure. The study also examined the relation of personal empowerment with personal recovery (*r* = .67) and psychiatric symptoms (*r* = -.39). The shortened version, which is detailed in the method section in this article, produced better confirmatory fit statistics (*CFI* =0.835, *GFI* =0.878, *RMSEA* = .070, and *NNFI* =0.835), and maintained good reliability for the overall scale (α = .82) in terms of internal consistency. However, the subscales scores varied from a modest to an excellent internal consistency (esteem-efficacy, α = .82; power and powerlessness, α = .59; community activism and autonomy, α = .59; optimism and control over future, α = .45; righteous anger, α = .64).

PW Corrigan, D Faber, F Rashid and M Leary [25], with a group of individuals released from an inpatient service and from a partial hospitalization programme (*N* = 35), used an earlier unpublished version of the ES with seven factors to test a model that included two super ordinate factors: the dimensions of self- and community orientation to empowerment. Previous reliability analysis for the subscales showed a low righteous anger (α = .38) and this factor was removed from the analysis. The remaining subscales showed good reliability (α > .75): self-efficacy, powerlessness, self-esteem, optimism and control over the future and group/community action. The Empowerment Scale was also tested with participants (*N* =283) from an outpatient public mental health service bySA Wowra and R McCarter [20] that confirmed its reliability (*α* = .85) and its five-factor model. Interestingly, the authors found that respondents with full-time jobs and college experience scored higher in the overall empowerment.

A review of existing literature showed the 28-item version of the Empowerment Scale had also been translated and subjected to psychometric analysis across the counties of Sweden, Japan and the Netherlands [21–24]. L Hansson and T Björkman [21]highlighted the validity of the ES in the course of a follow-up study among participants from case management services in Sweden (*N* = 92) and found a very satisfactory Cronbach’s alpha for the overall scale’s internal consistency (*α* = .84), and of .64 to .90 coefficients levels for the subscales except for the power-powerlessness subscale (*α* = .45). The study also supported the second-order factor structure proposed in previous research [25]. The Swedish scale presented significant and positive association with quality of life, size and quality of social network and psychological functioning and associated negatively with psychiatric symptoms, needs for care, and with the negative stigmatizing attitudes.

The Empowerment Scale was also adapted for the Japanese context [24] and used in a second study with 72 respondents from one mental health day and vocational service [22] to determine their level of empowerment and to examine the ES results with social adjustment and attitudes towards negative circumstances. In both studies, significant correlations between the factors and the overall score were found, except in the case of righteous anger. Likewise, this subscale had inverse correlations with power and with optimism for the future. S Yamada and K Suzuki [22] accounted for the significance of the righteous anger subscale in the measure. S Castelein, M van der Gaag, R Bruggeman, JT van Busschbach and D Wiersma [23], in an outpatient service in the Netherlands (*N* = 50) compared the properties of three empowerment measures, including the Empowerment Scale. They reported satisfactory internal consistency for the ES (α = .82) and its sensitivity to the symptom scores.

### Aim of the study

Considering the need for the development of outcome measures to evaluate empowering interventions with people who experience mental illness, the current study aims to assess the reliability and validity of the Portuguese version of the Empowerment Scale. Regarding the construct validity, it was also hypothesized that empowerment would be positively correlated with personal recovery and negatively correlated with psychiatric symptoms.

## Method

### Study design and data collection

The present validation study was conducted as part of a cross-sectional research project on mental health recovery and community integration of people who have experienced mental illness [31, 32]. The participants were invited by letter to participate by five Portuguese non-profit, community mental health organizations with similar programmes. All research settings in the study were *community centre* and/or *socio-occupational forum* acting under the same policy regulation for the delivery of psychosocial rehabilitation and community programmes in Portugal. Six organizations operating in urban environments were invited to take part in the study, as follows: one from the north of the country (Oporto), four from the Lisbon area or surrounding neighbourhoods (the region with the largest number of organizations including the two with the largest number of users) and, one from the south region (Faro). One of these settings (the one from the north area) was not included later in the study due to the small number of participants that agreed to participate. Most of the data collection occurred during 2012.

The study sample was one of convenience and respondents were assigned according to the criteria of age (≥18 years), personal history of psychiatric treatment or hospitalization, participation in the rehabilitation/community programme (≥3 months), and their willingness to participate in the study. They provided their written informed consent, demographic information and filled in three measures regarding their personal empowerment, recovery in mental health and psychiatric symptom distress. The questionnaires were filled in during individual face-to-face interviews. Items were self-rated by the study participants but could be read by the interviewer when deemed necessary.

### Measures

The assessment protocol for the present psychometric study included a questionnaire about the demographic background of the respondents and valid measures regarding the personal empowerment, individual recovery, and psychiatric symptoms described below.

The Empowerment Scale developed by ES Rogers, J Chamberlin, ML Ellison and T Crean [18], is a tool intended to measure subjective accounts of personal empowerment among users of mental health services, on a 4-point Likert scale ranging from “strongly agree” to “strongly disagree”. A high score on an ES factor score represents a high endorsement of that factor. The current study used the 25-item shortened version[19],which has reported good internal consistency (α = .82) and is composed of items such as “I am usually confident about decisions I make”, “Working with others in my community can help to change things for the better” or, “I can pretty much determine what will happen in my life”. The ES factors tap into five domains of self-esteem and efficacy (esteem subscale), power-powerlessness relations (power subscale) optimism and control over the future, (optimism subscale), righteous anger (anger subscale), and community activism and autonomy (activism subscale).

To analyse concurrent validity of the empowerment measure the Portuguese short version of the 24-item Recovery Assessment Scale (*RAS*) (MF Jorge-Monteiro and JH Ornelas: Participatory translation and validity of the Portuguese Recovery Assessment Scale, under review was used). The RAS resulted from the a longer 41-item scale [33, 34] and its items take into account domains such as personal confidence, hope for the future and attaining personal objectives, management strategies for personal well-being and having a life beyond illness or its symptoms. The RAS is considered to have strong psychometric properties.PW Corrigan, M Salzer, RO Ralph, Y Sangster and L Keck [33]reported excellent internal consistency (α = .93) and test-retest reliability (*r* = .88) for the original scale. The Portuguese RAS was subjected to a rigorous process of participatory translation and adaptation and also presented excellent level of internal consistency (α = .90) and good Cronbach’s coefficient alpha for the subscales (> .75).

The ES was also tested against the Colorado Symptom Index (*CSI*), which is a measure of psychiatric symptom frequency within a temporal frame [35]. In the current study we used the brief 14-item version, in which the participant rates the frequency of symptoms experienced in the past month according to a 5-point Likert scale (1- Not at all, 5- At least every day). KJ Conrad, JR Yagelka, MD Matters, AR Rich, V Williams and M Buchanan [36]found excellent internal consistency (α = .90) and test-retest reliability (*r* = .79). The CSI includes statements such as “In the past month, how often have you felt nervous, tense, worried, frustrated, or afraid?”, “In the past month, how often did you have problems thinking too fast (thoughts racing)?”

### Procedure and statistical analysis

For the ES scale adaptation, the English version was translated into Portuguese by two researchers, who were also community mental health practitioners knowledgeable in empowerment theory and practice. The intermediate proposal was then translated back into English by a bilingual translator following the usual procedure combined with a debriefing meeting between researchers and translator about the accurateness of statement wording in order to achieve the Portuguese ES version.

Construct validity of the ES was determined with confirmatory factor analysis [37] using a maximum likelihood estimation method to test the factorial validity of the measure. The model adequacy was analysed by the Comparative Fit Index (*CFI* > .90), Tucker-Lewis coefficient (*TLI* > .90), Goodness of Fit Index (*GFI* > .90), Root-Mean Square Error of Approximation (*RMSEA* < .05 with 90% *CI* < .10),Parsimony CFI (*PCFI* > .80) fit indices and; the χ2 statistics (χ*2/df* <5) which are acceptable indices of fit and were used to determine the adequacy of the model of measure [38, 39]. The parameter estimates of the refined model were also examined for significance level (>2.56; *p* 0.01) [40, 41]. Reliability was also assessed with Cronbach’s alpha coefficient for internal consistency, for the overall scale and its subscales. Distribution properties of ES items were evaluated with skewness, kurtosis and, with multivariate kurtosis coefficients. The convergent and discriminant validity of the Portuguese ES were also tested using bivariate Pearson correlation coefficients. Background characteristics of the participants were examined with independent sample t-tests and chi square tests to assess homogeneity among the sample of participants across the settings. The missing values (<5% of data) were previously substituted for their respective means in the cases of less than 3missing values per participant. All analyses were performed using the AMOS v.20 and SPSS v.19 statistical packages.

### Ethical considerations

The study protocol was previously subjected to approval by the administrative bodies of those CMHOs which had been independently granted permission for the data collection. The participants received oral and written information about the study’s purpose and about their rights. The respondents’ anonymity was ensured and participation in the study was voluntary. All of them received a 6 Euro incentive for participation in the larger cross-sectional study.

## Results

### Participants

The respondents comprised 213 participants using psychosocial and community support programmes from five community mental health organizations. Participants were aged between 19 to 74 years (*M* =41.57 ± 10.35), 143 were men (67.1%). The self-reported number of psychiatric hospitalizations varied from no hospitalization at all to 30 times during their lifetime (*M* =2.29 ± 3.99). Background demographic data described in Table 1 provides a detailed description of the study participants.

**Table 1:** **Background characteristics of participants (N =213)**

Characteristic	***n*** (%)	Characteristic	***n*** (%)
**Psychiatric diagnosis**		**Education (attended)**	
Schizophrenia	125 (58.7)	9 yrs	111 (52.1)
Bipolar disorder	33 (15.5)	12 yrs	56 (26.3)
Depression	23 (10.8)	Higher Ed.	41 (19.3)
Other	30 (14.1)	Total	212 (99.5)
Total	211 (99.1)	**Employment status**	
**Age, ranges**		Employed	8 (3.8)
<30 yrs	31 (14.6)	Unemployed	66 (31.0)
30-39 yrs	55 (25.8)	Retired	44 (20.7)
40-49 yrs	81 (38.0)	Social security	72 (33.8)
50-59 yrs	38 (17.8)	Vocational Trainee	18 (8.5)
≥60 yrs	8 (3.8)	Volunteer	2 (1.4)
Total	213 (100.0)	Total	213 (100.0)
**Participation in programme, ranges**		**Supported employment**	
<6 months	18 (8.5)	Yes	29 (13.6)
6 m – 2 yrs	55 (25.8)	No	184 (86.4)
3 yrs – 5 yrs	57 (26.8)	Total	213 (100.0)
6 yrs – 10 yrs	46 (21.6)	**Live with**	
>10 yrs	37 (17.4)	Alone	29 (13.6)
Total	213 (100.0)	Family	112 (52.6)
		Partner/Spouse	11 (5.2)
		Others (no family)	54 (25.4)
		Total	213 (100.0)
**Number psychiatric hospitalizations**		**Independent living**	
No hospitalization	51 (23.9)	Yes	17 (8.0)
1-2	70 (32.8)	No	196 (92.0)
3-5	55 (25.9)	Total	213 (100.0)
6-9	22 (10.4)	**Group Home**	
> 10	8 (3.8)	Yes	48 (22.5)
Total	206 (96.7)	No	165 (77.5)
**Marital status**		Total	213 (100.0)
Single	176 (82.6)	**Distribution by CMHO**	
Married/Relationship	11 (5.2)	CMHO-A	93 (43.7)
Divorced/Widowed/	26 (12.2)	CCMO-B	25 (11.7)
Total	213 (100.0)	CMHO-C	30 (14.1)
		CMHO-D	35 (16.4)
		CMHO-E	30 (14.1)

As the sampling procedure was one of convenience, participants were divided into two groups to observe their equivalence across the study settings as regards age, gender, symptoms and psychiatric diagnosis (48.7% self-reported schizophrenia). One group was composed of participants from CMHO-A with the larger number of participants (*n* = 93) and compared with participants from the other four organizations each with a smaller number of participants (*n* = 120), consequently resulting in two approximately equal halves. Results from independent-samples t-test showed that statistically significant differences were not found between the groups in terms of age *t* (211) = .94, *p* = .348 and the level of reported symptoms *t* (211) = -1.19, *p* = .232. Similarly, were observed non-significant associations in terms of gender *X*^2^ (1, *N* = 213) = .21, *p* = .65 and of diagnosis of schizophrenia *X*^2^ (1, *N* = 213) = .01, *p* = .91 from the chi square statistic. The group comparison allowed ascertaining the quality of the sample for current analysis in terms of background characteristics.

### Construct-related validity and reliability

The results of the confirmatory factor analysis to examine the ES construct validity suggested a fair fit of the five-factor model with 25 items, identified as Model 1 for the purpose of this study (Table 2) [38, 39]. The reliability analysis on the 25-item ES estimated an overall satisfactory internal consistency with Cronbach’s alpha coefficient level of .76. Considering the outcome data, subsequent analysis was conducted to improve the model’s fit and determine a better adjustment with this sample of participants.

**Table 2:** **Model fit statistics from CFA for Portuguese version of the ES**

	***X*** ^***2***^ ***/df***	***CFI***	***TLI***	***GFI***	***RMSEA***	***PCFI***
Model 1	2.210	.780	.751	.813	.076	.689
Model 2	1.779	.896	.876	.886	.061	.750

The ES model was refined according to the observed item factor loadings (< .45 in the current study), and the modification indices greater than11 (p < .001) [38]. Five items (“People have more power if they join together as a group”, “Most of the misfortunes in my life were due to bad luck”, “Experts are in the best position to decide what people should do or learn”, “When I am unsure about something, I usually go along with the rest of the group”; and “I feel I have a number of good qualities” items 2, 7, 15, 20 and 23 respectively) were excluded from the model because they presented a low factor weight in their respective first-order factor.

Model 2 of the ES had co-variances/correlated errors between the item “I am usually confident about the decisions I make” and the item “I feel I am a person of worth, at least on an equal basis with others” (-.32) from the esteem factor. Model 2still did not attain the best standard for optimal quality. However, the refined model was retained for the current analysis as it confirmed a better goodness-of-fit with a χ*2/df* = 1.779 and fit indices of: *CFI* = .896, *TLI* = .876, *GFI* = .886, *PCFI* = .750, and *RMSEA* estimation of .061, 90% *CI* [.05; .07], which is considered to be reasonable, as current results were similar and somewhat higher than those of the original study [19]. All parameter estimates registered as significant at *p* =0.01 ranging between 2.81 and 9.42. Lower parameter estimates were found among the power and anger factors elements. Conversely, esteem/efficacy, optimism and, activism/autonomy factors presented higher parameter estimates levels.

All individual items loaded enough on the respective first-order factor, with their weights ranging from .48 to .74 (Table 3). The ES distribution properties were evaluated according to *skewness* and *kurtosis* of items’ frequency distribution. Items did not deviate from normal distribution (*sk* <3; *ku* <10), which enabled the chosen validity analysis. Multivariate kurtosis was also observed (*Kurtosis/c.r.* = 4.07). Nevertheless, the confirmatory method of maximum likelihood estimation used in this analysis, would be robust to non-normal distribution of data [38].

**Table 3:** **Descriptives, Item-total correlation, factor loadings, Cronbach’s alpha for the Portuguese version of ES**

Item/scales	Min-max	Mean	***SD***	α	Item-total correlation	Factor loadings ^a^	***SK***	***KU***
**Esteem and efficacy**		**3**	**.52**	**.87**				
ES04	1 - 4	3.04	.74		.61	.73	-.569	.373
ES05	1 - 4	2.92	.74		.52	.71	-.368	-.029
ES08	1 - 4	3.13	.72		.55	.67	-.583	.302
ES11	1 - 4	3.05	.68		.54	.60	-.515	.695
ES13	1 - 4	2.80	.76		.58	.73	-.354	.070
ES16	1 - 4	2.97	.73		.59	.64	-.464	.208
ES17	1 - 4	2.95	.72		.60	.67	-.392	.135
ES21	1 - 4	3.14	.72		.58	.64	-.672	.646
**Power – powerlessness**		**2.41**	**.73**	**.56**				
ES14	1 - 4	2.38	.92		.19	.74	-.040	-.893
ES19	1 - 4	2.44	.83		.13	.54	-.021	-.558
**Activism and autonomy**		**3.19**	**.49**	**.72**				
ES10	1 - 4	3.26	.68		.33	.60	-.781	.928
ES18	1 - 4	3.15	.73		.37	.56	-754	.764
ES22	1 - 4	2.96	.79		.20	.48	-.618	.240
ES24	1 - 4	3.22	.67		.47	.66	-.662	.167
ES25	1 - 4	3.32	.67		.37	.64	-.951	.167
**Optimism over future**		**2.72**	**.75**	**.52**				
ES01	1 - 4	2.59	.95		.40	.49	.067	-.965
ES12	1 - 4	2.84	.867		.56	.73	-.347	-.532
**Righteous anger**		**2.09**	**.61**	**.55**				
ES03	1 - 4	2.05	.83		.00	.51	.615	.028
ES06	1 - 4	2.19	.88		-.11	.62	.501	-.343
ES09	1 - 4	2.02	.79		-.05	.49	.665	.348
**Total**		**2.82**	**.35**	**.79**				

*Note.* ^a^obtained from the *CFA* for the adjusted model (Model 2).

The findings as regards item-total correlations, also presented in Table 3, varied from moderate to strong for items in the esteem and efficacy factor; moderate among community activism and autonomy and in optimism over future items; and appeared modest and poor in items from power-powerless and righteous anger, indicating that those items revealed a divergence from the total construct measure used in this study.

An overall empowerment mean score for the sample and for each factor/subscale was obtained by summing the scores of individual items and dividing by the total number of items (Table 3). The mean score for the total scale (*M* =2.82 ± .35) was similar to that of the original validation study and above the mid-point for the measure that indicates a high score outcome. The new model solution improved the overall ES reliability in terms of internal consistency (α = .79) as presented in Table 3. Cronbach’s alpha coefficient for the subscales ranged from fair to good (esteem, α = .87; power, α = .56; activism, α = .72; optimism, α = .52; and anger, α = .55).

The estimated bivariate correlations (*p* < .01) among the ES factors ranged from strong to fair (Table 4). Strong to moderate associations were found between the esteem factor and optimism over the future (*r* = .65) and community activism (*r* = .44); and between activism and optimism (*r* = .28). The power factor had poor inter-correlations with esteem (*r* = .20) and with anger (*r* = .01) factors, appearing as independent factors. Poor negative correlations were found between righteous anger and esteem (*r* = -.17) and with optimism (r = -.15) factors (*p* < .05). Non-significant correlations (*p* > .05) were also estimated between power and activism, and optimism factors; and between activism and anger. Bivariate correlations between the total score of the ES and its subscale scores presented very strong and moderate associations (esteem, *r* = .89; optimism, *r* = .70; activism, *r* = .63; power, *r* = .34) with the exception of the righteous anger factor with a non-significant estimation (*p* > .05, *r* = .09).

**Table 4:** **Pearson correlations among the ES first-order latent factors and with the external variables**

Scale	F1	F2	F3	F4	F5	RAS	CSI
F1. Esteem-efficacy							
F2. Power-powerlessness	.20						
F3. Community activism-autonomy	.44	-.09°					
F4. Optimism/control over future	.65	.16°	.28				
F5. Righteous anger	-.17^*^	.01	-.12^o^	-.15^*^			
RAS	.72	.12	.45	.51	-.11°		
CSI	-.21	-.35	.05^o^	-.13°	-.03°	-.22	
ES	.89	.34	.63	.70	.09°	.71	-.22

*Note.* All correlations are significant at *p* < .01 level, with the following exceptions: (*) - significant at *p* < .05 level and (°) – not significant (*p* > .05).

The Pearson’s correlation coefficients between the total scale and the RAS and the CSI measures are presented in Table 4. As hypothesized, estimates showed the Empowerment Scale strongly correlated with personal recovery (*r* = .71). The analysis also found moderate inverse correlations of the CSI psychiatric symptom index with the ES (*r* = -.22) and RAS (*r* = -.22) scores, suggesting that, although related, the empowerment and recovery scales are measuring something very different from manifestations of the illness.

A similar analysis was also conducted between the empowerment subscales and the external variables. The esteem (*r* = -.21) and power (*r* = -.35) subscales presented negative correlations with the CSI total score and non-significant correlations (*p* > .05) with optimism (*r* = -.13), activism (*r* = -.05) and with anger (*r* = -.03). The RAS overall score showed positive correlations (*p* < .01) with esteem (*r* = .72, optimism (*r* = .51); activism (*r* = .45) and power (*r* = .12) subscales; but non-significant (*p* > .05) correlation with righteous anger (*r* = -.11). The interpretation of these results needs to take into consideration the reported data on internal consistency for the subscales.

## Discussion

To the best of our knowledge, this is the first empirical study to use the 25-item Empowerment Scale short version [19]. The current study replicated the proposed five-factor model to assess reliability and validity of the Portuguese version with a sample of 213 participants from five representative CMHO psychosocial/community support programmes. Our respondents’ sample represents an important portion of users taking advantage of these services in Portugal. A report of the “Carta Social” (Social Chart) from 2012 showed that 800 people were supported by this type of programme. This data is available at a website from the Social Security Institute (http://www.cartasocial.pt/pdf/csocial2012.pdf).

Observing the findings from reviewed studies [10, 12, 25], they suggested the usefulness of empowerment as a psychological construct for people who experience mental illness in diverse clinical and social environments, as well as cultures. Thus, the processes of empowerment are relevant features in current mental health interventions [1, 28] and throughout worldwide mental health policy reform and transformation [2, 26]. Therefore, it is important to have construct-related validity of translated measures to facilitate the study and comparison of intervention efficacy across different countries’ mental health systems.

From our factorial validity analysis, in the face of preliminary unsatisfactory fit statistics which were nonetheless not so wrong as to be inadmissible, the ES model required its refinement to achieve a better and reasonable adjustment to the data. The adjusted model yielded a better 20-item solution [39]. The original validation study from ES Rogers, RO Ralph and MS Salzer [19], revealed similar quality issues.

Confirmatory factor analysis performance is impacted by circumstances that may affect the validity of outcome measurements such as the hypothesised model, the measurement instrument itself (e.g. number of items per latent factor and its feasibility), the sample size, multivariate normality and the parameter estimates [38, 40]. Concerning the theoretical model, the ES is a consumer-constructed scale strongly anchored in the mental health advocacy consumer movement [4, 18] and that background foundation is consistent and relevant for the ongoing transformative changes in mental health systems [2]. The measure is based on an empowerment definition that incorporates process components such as being hopeful, learning and thinking critically in terms of personal agency and efficacy and decision making, which are psychological-related dimensions; and group/community-oriented dimensions such as the relationship to the institutionalized power, including learning about expressing righteous anger, feeling part of a group, increased capacity to act, and effecting change in one’s community [4, 9, 14, 15]. Therefore, one may consider that the model under consideration captures essential empowerment domain criteria in the mental health field and in users’ experience.

Current factorial validity of the Portuguese version of ES revealed respect for non-severe violation of multivariate normality and presented reliable parameter estimates [40]. On the other hand, the refined measurement confirmed two latent factors (optimism and power) with less than three items in the model which maybe is considered an impairment in the performance of an outcome measure [42]. Also the five excluded items appeared to be theoretically related with personal empowerment developmental processes, excepting just two of them (“Most of the misfortunes in my life were due to bad luck”, and “I feel I have a number of good qualities”) that may not demonstrate an empowerment feature but just general qualities. According to current analysis, though consistent with empowerment theory, the excluded items may reflect a different portion of empowerment processes not sufficiently pertinent to the factors presented in the model [40, 43, 44].

The present study also found strong to moderate correlations between the overall empowerment scale and its subscales with the exception of the “anger” subscale, which showed no significant association. This non-significant correlation was also mirrored in the “anger” subscale item-total results, underlining the specificity of the variable within the measure. These factorial-related findings are close to what was found in other studies [19, 21, 22, 25] with few items systematically weighted at latent factors different from the original ones. S Yamada and K Suzuki [22] also highlighted the significance of the righteous anger subscale when applying the ES cross-culturally, namely how respondents perceive anger behaviours, attitudes and judgements. In our case, questions of how to translate “anger” and, “angry” into the Portuguese language, may be pertinent [42, 43].

L Hansson and T Björkman [21], considered that the inherent contradiction in subscale items, some addressing perceived power and others addressing perceived powerlessness may affect the internal consistency of the “power” subscale. In the present study, most items in those subscales presented lower item-total correlations although they still loaded enough in the respective factors and, coincidently most inversely-stated items dropped from the adjusted model. While aiming for response accuracy, that approach may have been somewhat confusing for the respondents [45].

For the current study with this sample of community mental health users, both circumstances, such as the comprehensiveness of the model of measure in terms of manifest and latent theoretical components across stages of personal empowerment and; the accurateness of the measurement in terms of some items inversely stated, the number of items per factor and the cultural or context specificity of the righteous anger factor, may have affected significantly ES factorial validity estimates [40, 44].

The exclusion of items is not considered a sufficient reason for model improvement, rather it had the purpose of finding the better adjustment of the model in relation to the data with the current sample of participants [38, 40, 41]. In order to obtain a stronger ES there is a need for further investigation to improve its less robust aspects. Issues of ES content validity should be substantiated with constituent involvement in qualitative methods. Small group with people who experienced mental illness at different stages and from diverse contexts of participation (*eg*. psychosocial, community integration supports and advocacy), are a way of exploring arenas of personal empowerment across mental health system settings. Content validity must also combine *thinking-aloud* with *verbal probing* techniques for cognitive item evaluation, particularly in the cases of “righteous anger” and “power-powerlessness” items to verify potential issues of lexical accuracy and cultural or contextual-related aspects [43, 45].

According to ES Rogers, RO Ralph and MS Salzer [19], empowerment is a construct that can be positively affected by settings characteristics and thus may be a modifiable psychological outcome in mental health. The construct-related validity is, therefore, a continuing process; it cannot be proved definitively [43]. This empowerment assumption is of relevance to its relation with the values and empowerment-recovery orientation of the current mental health systems as they change worldwide [2].

Another assumption of empowerment is that an individual does not have to display every quality specified by the definition because it is not an a defining “status” but rather a process of growth and change through participation [4, 15]. The results from our study demonstrated that the overall mean score for the sample was above the midpoint for the instrument, which indicates a high level of empowerment for the current study participants from community programmes.

In terms of reliability analysis, the refined ES achieved an overall satisfactory internal consistency level, which parallels the Rogers’ study. Consistent with factorial data, current results for the subscales’ internal consistency varied from good to excellent in the esteem-efficacy and activism components but less satisfactory for perceived power, optimism and anger subscales, as reported by Cronbach’s alpha correlation levels.

Likewise the authors of the original study [19], due to identical subscale reliability issues, proposed solely the use of the overall ES as a valid and reliable measure. The 20-item Portuguese ES also proved reliability for its use as an overall empowerment measurement which permitted the use of the conducted convergent and discriminant validity analysis with the concurrent measures of recovery and symptoms.

Consistent with conceptual and empirical assumptions, empowerment and recovery showed themselves to be strongly associated, as empowerment is considered an important mediator for mental health recovery [6, 8, 9, 27, 30]. The concurrent analysis for the overall scale confirmed the hypothesized results. Findings indicate that the ES is measuring a defined psychological construct that is qualitatively-related in the same direction with personal recovery; and inversely with manifest psychiatric symptoms. Our results were similar to those from the reviewed studies with the same or equivalent measures [19, 21, 25].

The current study determined satisfactory reliability of the overall ES for its use in community mental health organizations. Validity was also assessed and ensured by the convergent and discriminant analysis in terms of construct validity, being that the inputs of the factorial analysis highlighted the need for improvements to the model in order to achieve a stronger empowerment measurement in the context of the mental health system.

### Strengths and limitations

The current study is, to the best of our knowledge, the only psychometric study using the ES shortened version [19] with participants from community mental health organizations. While parameter estimates were reliable, with the sample size being an important condition performing structural equation modeling (number of cases per estimated parameter) the ratio of 4.17 needs to be reported here [38, 46]. Facing the scarcity of empowerment measures in the mental health field, the development of a reliable Portuguese measure of personal empowerment is a fundamental requirement for there to exist empowerment/recovery-oriented measures in the mental health services. The translated equivalent ES also fosters the capacity to compare results on empowerment across different countries. This study also added evidence of the need for future factorial evaluation of the ES scale.

## Conclusions

This study provided a unique empowerment outcome measure in the community mental health field in Portugal; it also represented a shift in the capacity to develop studies on empowerment across a number of countries. The performed validity analysis reported convergent and discriminant validity of the empowerment construct and underlined the need to improve its factorial-related validity. The authors also presented suggestions to address such a need. Despite the necessity for an enhanced measurement the findings indicate that the current overall ES is reliable for the use in the Portuguese community mental health field.
